# ISCEV guidelines for calibration and verification of stimuli and recording instruments (2023 update)

**DOI:** 10.1007/s10633-023-09932-z

**Published:** 2023-06-03

**Authors:** Daphne L. McCulloch, Michael Bach, Mitchell Brigell, Hoover Chan, Ruth Hamilton, Chris Hogg, J. Vernon Odom, Anthony G. Robson

**Affiliations:** 1 School of Optometry and Vision Science, University of Waterloo, Waterloo, ON Canada; 2 Eye Center, Medical Center – Faculty of Medicine, University of Freiburg, Freiburg, Germany; 3 Consultant, Belmont, MA USA; 4 Department of Ophthalmology, University of California, San Francisco, USA; 5 The Smith-Kettlewell Eye Research Institute, San Francisco, USA; 6 Department of Clinical Physics and Bioengineering, Royal Hospital for Children, NHS Greater Glasgow and Clyde, Glasgow, UK; 7 College of Medical, Veterinary and Life Sciences, University of Glasgow, Glasgow, UK; 8 Institute of Ophthalmology, University College London, London, UK; 9 Department of Ophthalmology and Visual Science, West Virginia University, Morgantown, WV USA; 10 Department of Neuroscience, West Virginia University, Morgantown, WV USA; 11 Department of Electrophysiology, Moorfields Eye Hospital, 162 City Road, London, EC1V2PD UK

**Keywords:** ISCEV guidelines, Calibration, Verification, Photometry, Clinical electrophysiology, Electroretinogram (ERG), Pattern ERG, Multifocal ERG, Visual evoked potential (VEP), Electro-oculogram (EOG)

## Abstract

This document developed by the International Society for Clinical Electrophysiology of Vision (ISCEV) provides guidance for calibration and verification of stimulus and recording systems specific to clinical electrophysiology of vision. This guideline provides additional information for those using ISCEV Standards and Extended protocols and supersedes earlier Guidelines. The ISCEV guidelines for calibration and verification of stimuli and recording instruments (2023 update) were approved by the ISCEV Board of Directors 01, March 2023.

## Introduction

### Background and purpose

Clinical electrophysiology of vision comprises non-invasive tests which assess visual function including different types of electroretinogram (ERG), visual evoked potential (VEP) and electro-oculogram (EOG) [1]. The International Society for Clinical Electrophysiology of Vision (ISCEV) establishes and regularly updates clinical Standards and approves extended protocols for clinical electrophysiology of vision tests, to promote reliable diagnosis and monitoring with comparable results across testing centres [https://iscev.wildapricot.org/standards]. ISCEV also publishes Guidelines that provide context for the ISCEV Standards and protocols. The ISCEV Guide to visual electrodiagnostic procedures [1] introduces the principles and common clinical applications of ISCEV Standard tests. These Guidelines to calibration and verification aim to provide concise information for practitioners and instrument manufacturers, for the calibration, measurement and verification of instruments and protocol settings; this updates and supersedes the 2003 Guidelines for calibration of stimulus and recording parameters used in clinical electrophysiology of vision [2].

ISCEV standards for the full-field ERG [3], EOG [4], VEP [5], pattern ERG (PERG) [6], multifocal ERG (mfERG) [7], as well as eight ISCEV extended protocols [8–15], specify stimulus and recording parameters. For all protocols, the electrical potentials of the retina or cortex evoked by visual stimuli are detected from surface electrodes, amplified, filtered and digitised, then measured, typically with averaging to extract the signal associated with the stimuli. As both stimulus and acquisition system characteristics have substantial effects on the waveform, peak time and amplitude of responses, meaningful interpretation requires stimulators and acquisition systems to be accurate. Regular verification and periodic calibration of stimulus and recording parameters is essential to ensure the reliability and stability of equipment and stimuli, for reliable sequential monitoring and comparison with reference data.

This document, including “Appendices 1–5”, provides guidance for calibration and verification of stimulus and recording systems specific to clinical electrophysiology of vision.

### Clarification of terms

The authors understand that the terms calibration, adjustment and verification have strict metrological definitions [16]. Given the importance of accessible Guidelines, we use the terms here with their more broadly understood but colloquial meaning. ‘Verification’ is used here to mean the process of measuring a parameter, for example flash strength, to ensure it remains within the tolerance specified by the appropriate Standard. ‘Adjustment’ is used here to mean the process of altering a parameter which has drifted beyond the measurement uncertainty of the instrument. ‘Calibration’ is the process which encompasses verification measurements and any required adjustments compared with a reference standard such as the calibration undertaken by manufacturers.

“Appendix 5” is an additional reference list selected by ISCEV members to provide broader information on topics such as physiologic amplifiers, signal processing, physiological band-pass filters, visual display systems and/or photometry. As for all other ISCEV Standards and Guidelines, this document does not contain safety standards, standards for clinical care and management, or advice for patient privacy or data protection; users of this Guideline must choose instruments approved for clinical use in their jurisdiction and follow local requirements for clinical safety, care, and data protection. This Guideline was adopted by the ISCEV Board of Directors as an official publication on 1 March 2023.

## Stimulus calibration and verification

### Background

Three types of stimuli are used in ISCEV Standard tests and extended protocols. Luminance flash stimuli provide uniform stimulation across retina; these are used in combination with full-field, uniform, steady backgrounds for light-adapted testing. Pattern stimuli for ISCEV Standard tests are high contrast black-and-white checkerboards presented as either contrast reversal or as onset/offset from a uniform grey field that matches the space-averaged luminance and colour of the checkerboard. Stimuli for multifocal testing comprise an array of hexagonal elements that change rapidly between dark and light (on or off) driven by a predetermined pseudorandom binary sequence (m-sequence).

The light stimuli used in ISCEV protocols are measured using photometric quantities, a system that weights electromagnetic radiation by the nominal spectral sensitivity of a standard human observer. Luminance is the photometric quantity pertinent to extended sources such as a visual display screen and its pattern elements and to uniform backgrounds presented in an integrating sphere (ganzfeld). For brief flashes, the pertinent quantity is time-integrated luminance. The strength of ISCEV stimuli are specified in photopic units which are based on the relative sensitivity of the light-adapted cone system; in addition, for dark-adapted recordings of full-field ERGs, both photopic and scotopic units may be specified. Current ISCEV Standards stipulate achromatic stimuli. Pattern stimuli are specified as black-and-white; brief flashes and backgrounds are specified as visibly white, with a scotopic to photopic ratio of approximately 2.5 (see [3]). Chromatic (non-white) flashes and backgrounds are used in several of the ISCEV extended ERG protocols [8–10, 13]. When testing under dark-adapted, rod dominated conditions, it is necessary to comply with scotopic specifications for both achromatic and chromatic stimuli because the scotopic to photopic ratios of stimuli vary markedly with the spectral content of the light.

### Visual stimulus generators

Flash and pattern stimulus generators for clinical electrophysiology of vision testing should be capable of matching the specifications of the Standard protocols and preferably allow generation of a broader range of stimuli to accommodate ISCEV extended protocols and other, customised testing. Stimuli should be calibrated and remain stable over time, with regular verification. Adjustments are required when a visual stimulus generator fails to provide a stable output. The limits specified by the various Standards are designed to be inclusive of a range of instruments and established laboratory reference data; within laboratories instruments should be adjusted to remain within the measurement uncertainty of the instruments. We encourage manufacturers to include automated protocols for verification and adjustment and for laboratories to use these frequently (i.e. daily or before each patient) when these are available.

Most manufacturers offer stimulus calibration with a known measurement standard as part of ongoing maintenance or service contracts. If practitioners do not undertake their own photometric calibrations, they should obtain manufacturer calibrations. An earlier version of this Guideline recommended verification of calibrations at a maximum interval of 6 months [2]. However, stability of stimuli is highly dependent on the technology used. The current maximum intervals for verification based on technology are tabulated in “Appendix 1”. More frequent checks may be required to check stability if values change substantially between successive measurements. The onus is on practitioners to ensure adequate verification and any necessary calibration of stimuli and to interpret recordings with appropriate consideration of stimulus accuracy and stability.

When automated systems are not available, measurement for verification and adjustment, requires a photometer(s) capable of measuring the luminance of both extended sources, including small areas (pattern elements) and the time-integrated luminance of brief flashes. The photometer should be capable of accurate measurement of low luminance levels, e.g. the pattern VEP dark check of approximately 0.1 cd m^−2^. It should be equipped to measure both photopic and scotopic quantities. The photometer field of view should be several degrees and preferably tens of degrees to measure ganzfeld stimuli. The time-integrating function must be capable of measuring a single brief flash as well as multiple brief flashes over several seconds and should be sufficiently sensitive to measure weak flashes (0.01 phot cd s m^−2^, 0.025 scot cd s m^−2^). Photometers require calibration, typically annually, which should use a source traceable to an international standard.

### Measurement of luminance stimuli

For continuously lit or reflecting sources such as display screens and ganzfeld backgrounds, the appropriate measure is luminance (units cd m^−2^). This is a measure of the steady-state luminance level of the surface. For brief flashes, the appropriate measure is time-integrated luminance (units cd s m^−2^). This is a measure of the total luminance delivered by the flash. For clinical electrophysiology of vision, brief flashes range from less than 100 microseconds for discharge lamps up to 5 ms for LEDs and other sources. “Appendix 2” describes methods for measuring brief flashes, flicker stimuli and backgrounds used in light-adapted full-field ERGs, flash VEPs and EOG tests.

### Measurement of pattern stimuli

Visual display units (VDUs) or other optical imaging systems generate pattern stimuli used in clinical electrophysiological tests, including VEPs, PERGs and mfERGs. “Appendix 1” lists the main types of VDUs with information about their application to clinical electrophysiology of vision and calibration frequency. Pattern stimuli require calibration and verification of their mean luminance, contrast, pattern element size and the field size.

*Mean luminance:* The mean luminance is of the utmost importance for pattern stimuli as it affects amplitudes and peak times, particularly for PERGs and mfERGs. Mean luminance is expressed as:

1 $$ \frac{{L_{\max } + L_{\min } }}{2} $$

where *L*_max_ and *L*_min_ are luminances of light and dark pattern elements, respectively, when these are equal in size and duty cycle (relative time of presentation). The ISCEV Standards require mean luminances of 40–60 cd m^−2^ for the VEP [5], > 40 cd m^−2^ for the PERG [6] and > 50 cd m^−2^ for the mfERG [7].

*Luminance transients:* Pattern reversal or pattern onset/offset stimuli must not have transient changes in mean space-averaged luminance of the whole display. Pattern onset/offset stimuli are particularly prone to unwanted luminance artifacts, where the uniform grey field does not match the patterned field in mean space-averaged luminance. Any transient or step change in luminance with each pattern change creates a luminance artifact that will contaminate the pattern response with a luminance response. A qualitative method of checking for luminance transients can be achieved by observation. With room lights off to enhance sensitivity, a sheet of white paper is held in front of, and parallel to, the plane of the stimulus at a distance of approximately 30 cm and viewed by an observer standing behind the display while the stimulus is delivered. The diffused reflection of the display on the paper should appear as a constant brightness. If any change is perceived, the stimulus must be adjusted to remove it.

*Contrast:* The Michelson contrast of pattern stimuli is defined as:

2 $$ {\text{Contrast}}\,(\%) = \frac{{L_{\max } - L_{\min } }}{{L_{\max } + L_{\min } }} \times 100 $$

where *L*_max_ and *L*_min_ are the luminances of the light and dark elements, respectively. Thus, contrast ranges from 0% for a homogeneous field to 100% when the dark checks have a luminance of zero. Although ISCEV Standard VEPs require a minimum contrast of 80% [5], VEP waveforms change very little for contrast levels above approximately 50%. However, mfERG and PERG amplitudes increase linearly with contrast so that stable, high levels of contrast are critical; minimum contrast levels are 90% for mfERGs and 80% for PERGs. It is advisable to protect manual controls for ‘brightness’ and ‘contrast’ to prevent accidental changes. Methods for measurement of pattern mean luminance and contrast are presented in “Appendix 3”.

*Element and field size:* Pattern evoked potentials show spatial tuning and thus are affected by the size (angular subtense) of the pattern elements. For guidance, a pattern element of 1 cm subtends approximately 1° (60 min of arc) at a viewing distance of 57 cm. The elements in checkerboard stimuli are specified by check width in degrees or minutes of arc. The hexagons in mfERG stimuli are scaled as specified in the mfERG standard with a smaller central hexagonal element surrounded by progressively larger elements. The field sizes for pattern stimuli are specified by the angular subtense of both height and width. Procedures for calculating visual angles for fixed viewing distances and for choosing a viewing distance to obtain a desired visual angle are given in “Appendix 3”.

### Triggers and temporal measurements

*Stimulus frequency:* Electronic instruments typically use precise crystal oscillators to regulate timing. These are accurate within nanoseconds and more accurate than physiologically relevant values. Thus, temporal frequency of stimulus presentation rarely requires validation and typically does not change within an instrument. It is important, however, that stimulus presentation is synchronised with a trigger for signal averaging to be effective and for the accurate measurement of peak times.

*Stimulus duration, onset latency and display rise times:* ISCEV stimuli vary from very brief flashes such as those from discharge lamps lasting a few nanoseconds to patterns that are sustained for half a second or more. Measurements from stimulus onset to the peaks in the response waveform are typically the most sensitive and specific diagnostic measures in clinical electrophysiology. ISCEV protocols presume that the trigger and the measurement point for stimulus onset are coincident; any delays introduced by the hardware or software of an instrument should be corrected before reporting peak times.

‘Stimulus onset’ is not instantaneous. Displays differ in the time taken to reach peak luminance (display rise time). Although CRT screens have rapid onset (within 1 ms) the onset is not simultaneous across the screen; the stimulus trigger for VEPs should be set at the midpoint of the raster or compensation made for the delay [5]. For PERGs the convention based on the 2013 ISCEV Standard is to set the trigger at the beginning of the raster scan or to note any differences [6]. For other displays (“Appendix 1”), the default assumption is that the onset trigger is set at the midpoint of the onset; any deviations or corrections for onset triggers should be noted.

When offset time is relevant, such as for mfERG recording, it is important that the total response time (onset plus offset time) is considerably less than the duration of an m-sequence step (e.g. < 13.3 ms for a step duration of one frame at 75 Hz frame rate) [7]. The onus is on the manufacturers and users to ensure that the onset timing of the instruments in use match those of the normative data. Care should be taken when changing instruments to correct for any changes in the timing and triggering of stimuli.

## Acquisition systems

### Background

Electrophysiological signals produced by the retina and visual cortex typically have small amplitudes in comparison to unwanted sources such as electrical signals at the line frequency (50 or 60 Hz) or from electronic equipment (often 100 or 120 Hz) and physiological ‘noise’ generated by muscle activity, electrical activity in the brain and eye movements. Differential amplifiers are used to amplify the difference between the two inputs, rejecting signals common to both inputs. Electrodes placed near the relevant signal source, distant from it, and at an indifferent point are connected to the positive (active), negative (reference), and common (ground) inputs of the acquisition system, respectively. High amplitude artifacts, such as those generated by eye movements or blinks, are typically excluded from the signal on-line or post hoc using rejection criteria based on amplitude; for example, the PERG Standard [6] suggests a ± 100 µV rejection threshold. Stimulus-locked signal averaging increases the signal to noise ratio; the choice of the number of averages should be governed by signal to noise conditions and meet the requirements of the specific Standards and extended protocols.

The amplified signal is digitised into arrays of time and voltage values using an analogue-to-digital convertor, and processing such as filtering and averaging reduces the noise further. Sampling frequency and some filter characteristics are specified in ISCEV Standards and extended protocols. Analogue filters and digital emulations introduce frequency-dependent phase changes and can alter waveforms and peak times which should be considered if filter settings differ, for example between serial recordings or between a recording and reference data. Amplifier gain should be adjustable and should ensure high amplitude resolution, and avoid any nonlinearities at the extremes of the range which could distort signals (e.g. clipping of the peaks).

### Measurement of electrode impedance

*Electrode impedance:* Impedance^1^ is measured by passing a low amplitude (≤ 1 µA) alternating current (AC, 10–100 Hz) through the tissue between a pair of electrodes. Devices used to measure impedance of physiological electrodes in situ must be specifically designed to do so and approved for medical use. Impedance depends on frequency but typically varies little between 10 and 100 Hz, the frequency range of interest in most physiological recordings. DC ohmmeters must not be used as these typically apply relatively high currents and will polarise electrodes, resulting in unreliable measures. Each ISCEV standard and protocol specifies the upper limits of inter-electrode impedance differences; low, matched input impedances will improve the signal-to-noise ratio of response waveforms. These specifications are pertinent to ‘passive’ electrodes: no specifications are made for ‘active’ electrodes which incorporate pre-amplification, as they have less stringent impedance requirements.

### Measurement of amplification systems

Calibration and verification of amplifier gain is assessed by passing a known signal, with amplitude and timing in the expected range of the physiological signals (e.g. 1 µV to 1 mV), through the amplification system. The known signal should pass through the entire system, including any pre-amplifiers incorporated into the electrode connection box or the electrodes themselves. The amplitude of the output should closely resemble that of the input multiplied by the gain and adjusted for the effect of any applied filters. To undertake verification of the amplifiers, the signal generator should be specifically designed and calibrated to ensure accuracy, stability and to avoid damage to the amplifiers. (See “Appendix 4”).

Most manufacturers offer calibration of acquisition systems initially and as part of ongoing maintenance or service contracts. If practitioners do not undertake their own calibrations, they should obtain manufacturer calibrations. ISCEV encourages manufacturers to provide methods for practitioners to perform interim verification of the amplification systems, for example to investigate suspected malfunction. A useful check that all amplifier channels are working similarly is to pass an identical signal through each channel with identical settings. If amplifiers are not performing as expected, or if distortions of signals are observed, the equipment needs to be repaired.

Calibration of amplification and filtering should be performed as specified by the manufacturer based on the known stability of the system. If there are no specified intervals, we recommend a maximum interval of 1 year. The onus is on practitioners to perform or commission adequate calibration of the system.

## Appendix 1: Visual displays

The major types of visual display units (VDUs) for pattern stimulation are compared in the table below.

**Table:**

Display type	Brief technical description	Comments pertinent to visual electrophysiology	Frequency of verification
Cathode ray tube (CRT)^a^	Vacuum tube containing electron guns whose beams repeatedly scan the front of the tube to display images on a phosphorescent screen	No longer produced. Continuous pixel size is useful for some applications, e.g. for visual acuity estimation. Fine patterns lose contrast	At least every 6 months. More frequently for older models e.g. every month
Liquid crystal display (LCD) with thin film transistor (TFT)^b^	Image elements (pixels) are capacitors with an insulating liquid crystal layer between transparent conductive layers, driven by column and row transistor switches	Widely available and relatively inexpensive. Unacceptable luminance artifact with pattern changes unless specially adapted, e.g. by inserting flicker at every frame, modulating a banded backlight, using feedback or feed-forward adjustments	Annual. Or more frequently based on hours of use^b^
Plasma display	Pixels are phosphor lined cells that form plasma (gas of ions) when voltage is applied causing excitation of visible photons in the plasma	Capable of high luminances. Pixels ≈3 × larger than LCD	Annual
Organic light emitting diode (OLED) display	Panel of light-emitting diodes (LEDs) in which the electroluminescent layer is a film of organic compound that emits light in response to an electric current	Technology evolving. Has potential to optimise stimulus presentation. Some have ‘picture processing’ detrimental to precise stimulation	Annual or more frequently based on hours of use
Digital light processing (DLP) display	Projection technique—matrix of microscopic mirrors on a semiconductor chip oriented rapidly to reflect light either through the lens or onto a heat sink	Array of tiny mirrors, used for projection. Timing of the trigger is not straightforward	Annual if the source is LED; minimum of 6-monthly for Xenon lamp sources

^a^CRTs perform optimally with mean luminance between 25 and 100 cd m^−2^; nonlinearities can occur with L_max_ > 200 cd m^−2^. Luminance is typically greater in the centre than at the periphery. The current ISCEV mfERG Standard [7] requires less than a 15% difference between the centre and periphery of the stimulus array. For Standard pattern VEPs, which are less dependent on eccentric stimulus elements, the maximum acceptable difference is 30% [5]

^b^For example, Eizo monitors should be calibrated after 200–300 h of use [Color Management Resources Eizo] https://www.eizo.com/library/management/calibration/

## Appendix 2: Measurement of luminance stimuli^2^

### Measurement of flash stimuli

A method for measurement of the time-integrated luminance of brief, single-flash stimuli is summarised below.

I. Preparation
  - Turn on the photometer and allow sufficient time to stabilise.
  - Add an appropriate photopic or scotopic filters if indicated and select the relevant correction factor or calibration setting.
  - Set the photometer to time-integration mode to measure time-integrated luminance in units of cd s m^−2^.
  - Place the detector at the position occupied by the eye during a test.
II. Measurement
  - Darken the room and turn off fixation lights and any background lights or infra-red monitoring devices, e.g. lamps for cameras in the ganzfeld.
  - Zero the photometer.
  - Measure a single stimulus flash, noting the reading once stable. Repeat at least five times to ensure stability of measurements.
  - Select the median value as the flash luminance. If individual values differ by > 10%, consult the manufacturer.
  - Alternatively, deliver a series of a known number of flashes separated by at least 1 s, and divide the measurement by the number of flashes. This method does not provide a measure of inter-flash variability.

### Measurement of flicker stimuli

A method for measurement of rapidly presented flashes, such as the 30 Hz flicker stimulus is given below. To calibrate flicker stimuli, it is typically necessary to measure in integration mode over a known period of time. If the exact flicker frequency is known, the number of flashes delivered can also be known. If measuring flicker stimuli delivered by flash units based on discharge lamps, it is important to delay the start of measurement until after the output has stabilised: discharge lamp flashes typically are not full strength when operating at 30 Hz.

I. Preparation
  - Turn on the photometer to stabilise, as above; use an appropriate photopic filter.
  - Set the lamp to flicker and allow it to stabilise (typically > 1 s for discharge lamps / not necessary for LEDs with more consistent output).
  - Operate the photometer in time-integration mode.
II. Measurement
  - Measure the output for a known, fixed time.
  - Calculate the number of flashes delivered in the time interval and divide by the number of flashes to obtain the strength of individual flashes in the flicker stimulus.

### Measurement of background luminance

Luminance of extended backgrounds such as the full-field light-adapting background for light-adapted ERG testing are measured in photopic cd m^−2^ (also called Lux).

I. Preparation
  - Turn on the photometer to stabilise, as above; use an appropriate photopic filter.
  - Use an extended field if available (e.g. 10 deg).
  - Set the photometer to steady-state mode, measuring luminance in units of cd m^−2^.
  - Place the photometer at the location of the patient’s eye during testing.
II. Measurement
  - Darken the room, or match the room lighting to the level used during testing. Room lighting must not alter the luminance of the surface to be measured. Turn off any fixation light or infra-red source.
  - Zero the photometer, typically using a light-excluding cap.
  - Note the measurement once stable, and repeat at least twice more to ensure stability.
  - Take 5 measurements discard the high and low values and average the 3 remaining values. If measurements have drifted more than the measurement uncertainty of the instrument consult the manufacturer or replace the bulb if indicated and repeat the verification making any required adjustments.

## Appendix 3: Measurement of patterned stimuli

### Luminance of light and dark pattern elements

This method requires a spot photometer, also known as a luminance meter, equipped with optics for measurement over a small surface area and usually with a means of monitoring the region that is being measured. Some devices work by directly contacting the emissive surface or screen, others are focussed from a short distance away. Direct contact measurements will not incorporate any additional luminance contamination from room illumination.

I. Prepare the equipment
  - Turn on the photometer and allow sufficient time to stabilise.
  - Add an appropriate photopic filter if indicated and select the appropriate calibration factor.
  - Set the photometer to steady-state mode, to measure luminance in cd m^−2^
  - Display a stimulus with large pattern elements, e.g. 2 deg checks on the display
  - Slow, or if possible, stop, pattern alternation
II. Measurement
  - Adjust room lighting conditions to those used during testing.
  - Zero the photometer with the detector covered.
  - Where appropriate, focus the photometer optics.
  - Position the detector so that it is perpendicular to the screen and so that the measurement field is no more than half the size of the bright or dark element being measured.
  - Obtain stable measurement of light and dark elements at the centre of the screen, usually the four elements surrounding the centre.
  - Repeat each measurement at least twice more to ensure stability.
  - Repeat e) and f) for the four elements at the outer corners of the checkboard near the screen periphery.
III. Calculations
  - Calculate mean luminance using Eq. (1) with median values of light elements (*L*_max_) and dark elements (*L*_min_) at the centre of the screen.
  - Calculate mean luminance for elements at the periphery of the screen.
  - Calculate stimulus contrast using Eq. (2) with luminance values obtained from the centre of the stimulus.

If values differ from the measurement uncertainty of the instrument, consult the manufacturer, or perform the required adjustments as appropriate.

### Measurement of visual angles

Methods for calculating visual angle for pattern elements with fixed viewing conditions, for choosing a viewing distance to obtain a desired visual angle and for determining the field size are given below.

I. Measurement of pattern element size at a fixed viewing distance
  - Measure the size of 10 elements (e.g. 10 check widths, whether black or white) across the centre of the screen and divide by 10 to obtain the mean element size. Do this horizontally and vertically to verify symmetry, i.e. that checks are square.
  - Measure the distance from the patient’s eye to the centre of the screen.
  - Divide the element size by this viewing distance.
  - Determine the angle whose tangent is equal to this value by using the inverse tangent (tan^−1^);^3^ convert from degrees to minutes of arc (min) if necessary by multiplying by 60 (e.g. the checks in the Pattern ERG standard of 0.8° equals 48 min.) Check sizes given in ISCEV Standards refer to the width (equal to the height) of a check.^4^
II. Calculation of viewing distance for desired visual angle
  - Measure the size of 10 elements (e.g. 10 check widths, whether black or white) across the centre of the screen and divide by 10 to obtain the mean element size.
  - Look up the tangent of the desired visual angle of one element (i.e. tangent of the desired visual angle^3^).
  - Divide the mean element size measured (step a) by the tangent of the desired visual angle (step b) to obtain the viewing distance.^5^
III. Field size calculation
  - Measure the diameter of the stimulus area (width and height)
  - Measure the viewing distance from the patient’s eye to the centre of the screen.
  - Divide the diameter by this viewing distance.
  - Determine the angle whose tangent is equal to this value by using the inverse tangent (e.g. tan^−1^). Consult the relevant ISCEV Standard to verify that your field size and the ratio of width to height meets the requirements.

## Appendix 4: Verification of amplifiers for clinical recording

Verification requires a signal generator capable of producing low amplitude output in the physiological amplitude range (or a standard signal generator with precision attenuators). Differing signals will allow verification of different aspects of the acquisition system. Sinusoidal signal inputs allow detection of changes in amplification and filter settings, and any harmonic distortion. Simulated electrophysiologic signals can be used to determine the effects of the amplification system on measurable characteristics of the signal of interest. A square-wave pulse signal allows detection of any ‘ringing’ in response to an abrupt voltage change. Signal generators themselves will require regular calibration.

If amplifiers are not performing as expected, or if distortions of signals are observed, the equipment needs to be repaired. A useful check that all amplifier channels are working similarly is to pass an identical signal through each channel with identical settings.

A suggested method for amplifier verification is given below. Note that verification of amplification systems must not be done with a person connected to the system.

### Amplifier measurement method

I. Preparation
  - Switch on amplifiers and signal generator and allow to stabilise (typically a minimum of 15 min)
  - Connect signal generator to the amplifier’s electrode inputs
  - Generate signals (e.g. 1 µV to 1 mV).
II. Measurements
  - Acquire and measure simulated electrophysiologic calibration signals as for a patient recording. If signal averaging is used, the signal generator must trigger the data acquisition system.
  - Verify nominal filter bandpass by measuring multiple sinusoidal signal frequencies that begin below and extend above low-pass and high-pass filter settings, (± 3 dB).
  - Optionally verify the accuracy of the amplitude calibration and waveform by acquiring and measuring simulated electrophysiologic calibration signals without the filters used for clinical testing (i.e. maximum band-pass available, ‘open’ or no filter settings)
  - If values differ from nominal values to a degree that would make a clinically significant difference, consult the manufacturer.

## Appendix 5: Additional resources for the ISCEV guidelines for stimuli and recording instruments calibration and verification

This list was compiled by the ISCEV Calibration Committee in 2022 as a resource for topics beyond the scope of the guidelines but relevant to understanding clinical electrophysiology of vision and non-invasive recording methods. The list is headed by topic and arranged in chronological order by publication date.

These resources were recommended ISCEV members as instructive and accessible and/or authoritative and are provided for convenience; ISCEV has not vetted or endorsed this list.

*Bioelectric recording*:

Bureau International des Poids et Mesures (BIPM). JCGM 200:2012 International vocabulary of metrology—Basic and general concepts and associated terms (VIM) 3rd edition see https://www.bipm.org/documents/20126/2071204/JCGM_200_2012.pdf

Metting van Rijn, A.C., Peper, A. & Grimbergen, C.A. High-quality recording of bioelectric events. Part 1 Interference reduction, theory and practice. Med. Biol. Eng. Comput. 28, 389–397 (1990). 10.1007/BF02441961

Metting van Rijn, A.C., Peper, A. & Grimbergen, C.A. High-quality recording of bioelectric events. Part 2 Low-noise, low-power multichannel amplifier design. Med. Biol. Eng. Comput. 29, 433–440 (1991). 10.1007/BF02441666

Metting van Rijn, A.C., Peper, A. & Grimbergen, C.A. The isolation mode rejection ratio in bioelectric amplifiers. IEEE Transactions on Biomedical Engineering, vol. 38, no. 11, pp. 1154–1157, Nov. 1991, 10.1109/10.99079.

Nagel, J. H. Biopotential Amplifiers. In: The Biomedical Engineering Handbook: Second Edition. Ed. Joseph D. Bronzino. Boca Raton: CRC Press LLC, 2000 ISBN 084930461X

Prutchi D & Norris M. Bandpass selection for biopotential amplifiers. Chpt in Design and Development of Medical Electronic Instrumentation: Practical Perspective of the Design, Construction, and Test of Medical Devices. Wiley Interscience, New Jersey 2005 iSBN 0-471-67623-3

Tankisi H, Burke D, Cui L et al (2020). Standards of instrumentation of EMG. Clinical Neurophysiology 131(1) 243–258. 10.1016/j.clinph.2019.07.025.

*Photometry and Colorimetry*

Wyszecki G, Stiles WS (1982) Color Science: Concepts and methods, quantitative data and formulae. 2^nd^ Edition. John Wiley and Sons, New York.

Ryer D, (1997) The Light Measurement Handbook by Alexander D. International **Light** Technologies ISBN 0-9658356-9-3. Free download: https://www.intl-lighttech.com/light-measurement-handbook

Ohno Y. Radiometry and Photometry Review for Vision Optics. In: Handbook of Optics III, Second Edition. Editor Bass M, McGraw-Hill, New York (2001). ISBN 0-07-141478-9.

Palmer JM. Radiometry and Photometry: Units and Conversions. In: Handbook of Optics III, Second Edition. Editor Bass M, McGraw-Hill, New York (2001). ISBN 0-07-141478-9.

Makous W. Optics and photometry. In: Vision Research. A Practical Guide to Laboratory Methods, Eds Carpenter RHS & Robson JG. OUP 1999. ISBN 0 19 852319 X.

Robson JG. Light sources. In: Vision Research. A Practical Guide to Laboratory Methods, Eds Carpenter RHS & Robson JG. OUP 1999. ISBN 0 19 852319 X.

Sharpe LT, Stockman A, Jagla W, Jägle H (2005) A luminous efficiency function, V*(λ), for daylight adaptation. Journal of Vision 5:3–3. 10.1167/5.11.3

McCulloch DL, Hamilton R (2010) Essentials of photometry for clinical electrophysiology of vision. Doc Ophthalmol 121:77–84. 10.1007/s10633-010-9233-2

Blankenbach K. (2015) Introduction to Display Metrology. In: Chen J., Cranton W., Fihn M. (eds) Handbook of Visual Display Technology. Springer, Berlin, Heidelberg. 10.1007/978-3-642-35947-7_142-2

Goodman T. (2015) Light Emission and Photometry. In: Chen J., Cranton W., Fihn M. (eds) Handbook of Visual Display Technology. Springer, Berlin, Heidelberg. 10.1007/978-3-642-35947-7_19-2

Blankenbach K. (2015) Luminance, Contrast Ratio, and Gray Scale. In: Chen J., Cranton W., Fihn M. (eds) Handbook of Visual Display Technology. Springer, Berlin, Heidelberg. 10.1007/978-3-642-35947-7_143-2

Stockman A (2017) Luminosity functions. http://www.cvrl.org/lumindex.htm. Accessed 9 Sep 2019

Köhler R. Chpt 2: Photometric and Radiometric Quantities, In: Handbook of Applied Photometry, ed DeCusatis C, 1997, American Institute of Physics. ISBN 1-56396-416-3.

*Non-invasive visual bioelectric potentials and signal detection*

Fahle M, Bach M (2006) Origin of the visual evoked potentials. In: Heckenlively J, Arden G (eds) Principles and Practice of Clinical Electrophysiology of Vision. MIT Press, Cambridge, London, pp 207–234

## Footnotes

1 Impedance: denotes resistance at frequencies other than direct current (DC). At the relatively low frequencies used in clinical electrophysiology there is little quantitative difference between the impedance and resistance.
